# Acceptance of wearable technology for the early detection of dementia‐causing diseases: perspectives from the CODEC II cohort

**DOI:** 10.1002/alz.089597

**Published:** 2025-01-09

**Authors:** Sarah Wilson, Emily Beswick, Rachel Morrell, Sharandeep Bhogal, Clare Tolley, Tim Whitfield, Kieran Wing, Ríona Mc Ardle, Zuzana Walker, Sarah Slight

**Affiliations:** ^1^ Newcastle University, Newcastle Upon Tyne UK; ^2^ University College London, London UK; ^3^ Essex Partnership University NHS Foundation Trust, Essex UK; ^4^ Essex Partnership University NHS Foundation Trust, Wickford UK

## Abstract

**Background:**

With global dementia prevalence estimated to reach 139 million by 2050,^(1)^ early detection of dementia‐causing diseases is crucial for promoting preventative interventions. Wearable technologies have the potential to detect early signs; however, they need to be acceptable amongst users. We explored user’s perspectives on the acceptability of wearable devices.

**Method:**

A sub‐group of participants from a research cohort, Predictors of COgnitive DECline using digital devices (CODEC‐II), were recruited. Participants used four wearables (smartwatch, EEG headband, active and passive smartphone app) for two weeks every three‐months over a year. Semi‐structured interviews were conducted with participants after the first two weeks. A framework thematic analysis approach was used, assisted by N‐Vivo (QSR, version 14.23.2). The Intelligent Systems Technology Acceptance Model^(2)^ aided in refining key themes.

**Result:**

Twenty‐one participants were interviewed, including those with subjective cognitive decline (n=10), mild cognitive impairment (MCI) (n=7), dementia with Lewy bodies (n=1), and caregivers (n=3). Key themes included ease of use, data security, usefulness, intention of use. Many participants expressed low digital self‐efficacy, relying on the research team for support with technology setup and troubleshooting. Those with MCI needed more assistance with synching data, and experience difficulty with some cognitive testing games in the active smartphone app. Some participants expressed concerns surrounding the potential for someone to hack data generated from their device. Others questioned the accuracy of the study smartwatch, as they compared its outputs to their personal ones. Participants with professional or caregiving responsibilities described how their busy lifestyle hindered their ability to use the active smartphone app every day; this was not expressed by retired individuals.

**Conclusion:**

Acceptance of using different wearable technologies for the early detection of dementia‐causing diseases varied amongst participants. Researchers should consider the difficulty level of cognitive testing games for those with cognitive impairment. Future work must explore ways to support individuals with low digital self‐efficacy, ensuring digital health equity is supported.

**References**:

Prince et al., 2015, The global impact of dementia, World Alzheimer report

Vorm and Combs, 2022, Integrating transparency, trust, and acceptance: The intelligent systems technology acceptance model (ISTAM). International Journal of Human–Computer Interaction.

